# M. Liebig on the Preparation of Cyanide of Potassium

**Published:** 1842-10-08

**Authors:** 


*Preparation of Cyanide of Potassium.* By M. Liebig.—One of the best methods of preparing the cyanide of potassium, consists, as is known, in the decomposition of the ferro-cyanide of potassium at a red heat; but several objections attach to this process, one of which is that a loss takes place of a third of the cyanogen contained in the salt employed. This salt, formed of two atoms of cyanide of potassium, and one atom of cyanide of iron, undergoes no change from a red heat in the first member of the combination ; but the second is decomposed into carburet of iron, with disengagement of nitrogen. The carburet of iron which is formed absorbs the fused cyanide of potassium like a sponge, and we are obliged to have recourse to solvents, and alcohol in particular, to obtain the resulting cyanide of potassium without iron and without loss.

But as cyanide of potassium possesses properties which are found to be extremely valuable in effecting reduction and separation in chemical analyses, I have endeavoured to simplify its preparation.

If eight parts of ferro-cyanide of potassium be well dried by a slight calcination, on a hot iron plate ; if this be afterwards well mixed, in fine powder, with three parts of dry carbonate of potash, and the mixture thrown at once into a Hessian crucible, previously raised to a dull red heat, and this temperature maintained, the mixture will first melt into a brown magma, with rapid disengagement of gas ; in a few minutes afterwards, when the fluid mass has been heated to redness, the dark colour will be seen to become brighter, and by continuing the fusion the contents of the crucible will become of a clear amber yellow. If a warm glass rod be introduced from time to time, the portion which adheres to it, when withdrawn and allowed to solidify, will at first be of a brown colour; it will afterwards become yellow; and, lastly, at the end of the operation, the liquid which adheres to the glass rod will be clear and colourless like water, and will set into a crystalline mass of a brilliant white.

During the fusion, there will be seen floating in the fluid mass some brown flakes, which ultimately unite in the form of a sponge, and assume a clear gray colour. If the crucible be now taken from the fire, and allowed slightly to cool, it generally happens that this gray powder settles entirely to the bottom ; the deposition may be facilitated by agitating it once or twice with the glass rod. The hot and melted mass which swims on the surface may now be easily decanted into a warm porcelain capsule, without the least portion of the powder, which has settled to the bottom, passing with it.

The mass, thus separated from the iron by decantation, will contain two combinations. It will principally consist of cyanide of potassium ; the other combination will be cyanate of potash. These two will be found in the proportion of five atoms of cyanide of potassium to one of cyanate of potash.

The following changes take place during the fusion of ferro-cyanide of potassium with carbonate of potash :—

In the commencement of the fusion, the cyanide of iron of the ferro-cyanide of potassium is decomposed with the potash of the carbonate of potash into cyanide of potassium and carbonate of protoxide of iron, from which latter the cyanide of potassium, at a higher temperature, takes the whole of the oxygen. In consequence of this reduction there will be obtained cyanate of potash and pure metallic iron.

If we suppose the mixture to contain two atoms of ferro-cyanide of potassium, and two atoms of carbonate of potash, it will be represented by the following formula :—

Ferro-cyanide of Potassium. Carbonate of Potash.

$$Cy_{12}\ Fe_2\ K_4 \quad + \quad K_2\ O_2\ 2CO_2 = Cy_{12}\ Fe_2\ K_6\ O_2\ 2Co_2$$

And after the fusion we shall have—

| Cyanide of Potassium. | | Cyanate of Potash. | Iron. | Carbonic Acid. |
|---|---|---|---|---|
| $Cy_{13}\,K_5$ | + | $Cy_2\,O, KO$ | $Fe_2$ | $2\,Co_2$ |

We obtain from two atoms of ferro-cyanide of potassium, five atoms of cyanide of potassium; consequently one-fourth more than by the fusion of that salt alone at a red heat. The cyanate of potash, with which it will be mixed, will not interfere with any of its uses; its presence will be easily detected by saturating the cyanide of potassium with an acid: it will give rise, in fact, to an effervescence caused by the disengagement of carbonic acid, and there will be found in the solution an ammoniacal salt.

The explanation of the formation of cyanide of potassium under the conditions indicated is not quite clear, because the carbonate of protoxide of iron which is formed, is decomposed before the reduction of the iron and separation of carbonic acid into carbonic oxide and ferroso-ferric oxide; and it is at the expense of these that an indeterminate quantity (at the most that which is indicated in the preceding formula) of cyanate of potash is formed.

The remaining metallic iron, as well as the sides of the crucible, are covered with cyanide of potassium; the best way to recover this, is to dissolve it in hot water, and to heat the solution with a small quantity of sulphuret of iron, which will readily dissolve. The cyanide of potassium may be obtained from this solution by evaporation, in the state of ferro-cyanide; sulphuret of potassium will remain in the mother-water.—*Pharm. Journ., from Ann. der Chimie und Pharm.*

